# Diffusion-Weighted Imaging of Small Peritoneal Implants in “Potentially” Early-Stage Ovarian Cancer

**DOI:** 10.1155/2016/9254742

**Published:** 2016-02-28

**Authors:** Laretta Grabowska-Derlatka, Pawel Derlatka, Wojciech Szeszkowski, Andrzej Cieszanowski

**Affiliations:** ^1^2nd Department of Clinical Radiology, Medical University of Warsaw, Banacha 1a Street, 02-097 Warsaw, Poland; ^2^2nd Department of Obstetrics and Gynecology, Medical University of Warsaw, Karowa 2 Street, 00-315 Warsaw, Poland; ^3^Department of Radiology I, Maria Sklodowska-Curie Memorial Cancer Center, Institute of Oncology, Roentgena 5 Street, 02-781 Warsaw, Poland

## Abstract

*Introduction*. MRI is established modality for the diagnosis of ovarian malignancies. Advances in MRI technology, including DW imaging, could lead to the further increase in the sensitivity of MRI for the detection of peritoneal metastases. The aim of this study was to assess the accuracy of DW imaging for detection of peritoneal metastatic disease in patients suspected of having potentially early ovarian cancer and secondly to evaluate ADC values of peritoneal implants.* Materials and Methods*. The prospective study group consisted of 26 women with sonographic or/and CT diagnosis of suspected ovarian tumor. Based on the results of the above imaging, in none of them was extraovarian spread of disease or ascites recognized. All patients underwent MRI with DW imaging.* Results*. Overall, 18 extraovarian peritoneal lesions were found on DW images in 10 from 26 examined patients. All implants had diameter ≤10 mm. The presence of all lesions diagnosed by MRI was confirmed intraoperatively. Histopathologic findings in 17 proofs confirmed ovarian cancer. PPV was 94%. On all DW images (with* b* values of 0, 50, 100, 150, 200, 400, 800, and 1200 s/mm^2^) the mean signal intensities of peritoneal lesions were significantly higher than the mean signal intensities of normal adjacent tissue (*p* = 0.000001).

## 1. Introduction

About 3300 new cases of ovarian cancer are annually diagnosed in Poland of which 70% are advanced (stages III and IV) [1]. Currently, the standard treatment for early-stage ovarian cancer is primarily surgical management (with or without chemotherapy). According to the International Federation of Gynecology and Obstetrics (FIGO) guidelines, the optimal staging procedures for early ovarian cancer are abdominal hysterectomy, bilateral salpingo-oophorectomy, peritoneal biopsy, omentectomy, diaphragmatic scraping, bilateral pelvic, and para-aortic lymph node dissection [2].

The treatment of patients with advanced-stage ovarian cancer is based on debulking surgery and adjuvant chemotherapy. The surgeon aims at achieving a maximal possible cytoreduction [3]. Numerous studies have shown that the patients in whom the removal of all macroscopic lesions was possible have the best prognosis [4–6].

When the disease has spread intra-abdominally, complete surgical tumor debulking is increasingly difficult. In patients with massive peritoneal spread, the method of choice is neoadjuvant chemotherapy, followed by interval surgery [7]. The interpretation of peritoneal findings at preoperative imaging requires detailed knowledge of the complex peritoneal anatomic configuration and the directionality of peritoneal fluid flow [8].

Magnetic resonance imaging (MRI) is established modality for the diagnosis of ovarian malignancies. This technique may be also utilized to determine the extension of disease, including detection of peritoneal metastatic disease in these patients. Advances in MRI technology, including improvement of DW imaging technique, could lead to the further increase in the sensitivity of MRI for the detection of peritoneal metastases. Studies have recently investigated the efficacy of DW images to detect peritoneal implants in different pelvic and abdominal malignancies [9]. The results were for the most part promising; however there are still important issues which need to be addressed and resolved, including the lack of standardization of DW images acquisition (choice of different* b* values) and various methods of calculation of apparent diffusion coefficient (ADC).

The aim of this study was twofold: firstly, to assess the accuracy of DW imaging for detection of peritoneal metastatic disease in patients suspected of having potentially early ovarian cancer and secondly to evaluate ADC values of peritoneal implants.

## 2. Materials and Methods

### 2.1. Patient Population

The prospective study group consisted of 26 women aged from 34 to 67 years with transvaginal and transabdominal sonographic diagnosis of suspected ovarian tumor. All patients had elevated serum levels of CA 125 or CA 19-9. In eight patients additional computed tomography (CT) of abdomen and pelvis was performed. Based on the results of the above imaging, in none of them was extraovarian spread of disease or ascites recognized.

### 2.2. MR Imaging

All patients underwent MR imaging of the abdomen and pelvis at our institution. MR imaging examinations were performed in all 26 patients using a 1.5 T clinical whole-body MR system (MAGNETOM Avanto; Siemens AG, Erlangen, Germany) with the Spine Matrix coil and combined two Body Matrix coils for larger coverage.

MRI protocol for the detection of the abdominal and pelvic lesions contained turbo spin-echo (tse) T2-weighted images, fat-suppressed T2-weighted, T2-TIRM, DW EPI, and pre- and postcontrast dynamic 3D T1 GRE in transverse orientation. The details of the applied parameters of MR imaging are presented in Table 1.

Axial DWI images were acquired using the same multislice EPI sequence for all patients: 34 × 6 mm slices (abdominal part) and 30 × 6 mm slices (pelvic part); 380 × 380 mm FoV; 128 × 96 matrix; TR = 3800 ms; TE = 73 ms; with diffusion weightings of 0, 50, 100, 150, 200, 400, 800, and 1200 s/mm^2^.

In all patients Gadobutrol (Gadovist, Bayer Schering, Berlin, Germany) was administered, at a dose of 0.1 mL/kg bodyweight, immediately followed by a bolus of 20 mL of physiological saline (NaCl 0.9%).

### 2.3. Image Analysis

Regions of interest were outlined in Multimodality Workplace Station (Siemens Medical Solution, Erlangen, Germany) by a genitourinary radiologist (with experience in pelvic MR imaging), who documented the number and location of peritoneal metastases on DW images and ADC maps. The number and location of metastatic peritoneal implants were confirmed intraoperatively and compared with MRI findings.

Subsequently, freehand regions of interest (ROI) were drawn on the ADC and all *b* values DWI images by using the T2-weighted images for guidance. ROI included largest possible part of the lesion, avoiding partial volume effect, areas of necrosis, and artifacts. Then ROI was copied and pasted from DWI image to corresponding ADC map and the measurement on ADC map was recorded. ADC was measured twice for each lesion and these measurements were averaged. Separate ADC measurements were performed for adjacent, normal tissues (e.g., liver, small bowel).

ADC values were calculated by monoexponential regression with the following formula: *S* = *S*_0_  ·  exp(−*b*  ·  ADC), where *S* is the signal intensity after application of the diffusion gradient and *S*_0_ is the signal intensity at *b* = 0 s/mm^2^. Eight* b* values were applied for ADC calculation.

The number and location of metastatic peritoneal implants were confirmed intraoperatively and compared with MRI findings.

The reference standard for the diagnosis was histopathologic proof obtained intraoperatively.

### 2.4. Statistical Analysis

All statistical analyses were performed using the STATISTICA ver.12 (Statsoft) software package. Comparisons of mean ADC and mean *b* values images (*b* 50, 100, 150, 200, 400, 800, and 1200 s/mm^2^) between peritoneal implants and normal surrounding tissue were analyzed using an unpaired *t*-test; ROC curve analysis *p* < 0.05 indicated a statistically significant difference.

## 3. Results

Overall, 18 extraovarian peritoneal lesions were found on DW images in 10 from 26 examined patients. The location of these lesions is presented in Table 2. All implants had diameter of 10 mm or less. The presence of all lesions diagnosed by MRI was confirmed intraoperatively. Histopathologic findings in 17 proofs confirmed ovarian cancer (13 serous, 3 mucinous, 1 endometrioid). One proof confirmed chronic inflammatory lymph node of hilus of the liver. No additional peritoneal implants were identified during surgical exploration; therefore positive predictive value (PPV) and negative predictive value (NPV) of DW imaging for the detection of metastatic peritoneal disease were, respectively, 94% and 100%.

**Figure 1:**
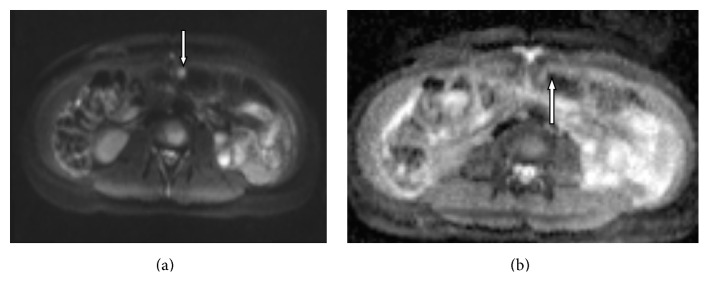
(a) DW image (*b* = 800 s/mm^2^) shows the small implant with high signal intensity (arrow) on transverse colon serosa. (b) On an ADC map, the implant demonstrates restricted diffusion (arrow).

**Figure 2:**
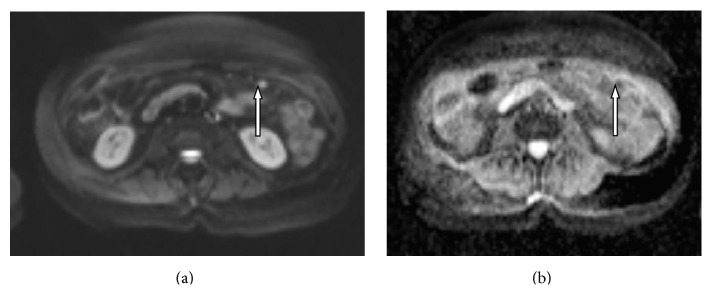
(a) DW image (*b* = 800 s/mm^2^) shows the small implant with high signal intensity (arrow) in omentum. (b) An ADC map, the implant demonstrates restricted diffusion (arrow).

On all DW images (with* b* values of 0, 50, 100, 150, 200, 400, 800, and 1200 s/mm^2^) the mean signal intensities of peritoneal lesions were significantly higher than the mean signal intensities of normal adjacent tissue (*p* = 0.000001). Mean ADC values of peritoneal lesions were significantly lower than those of adjacent tissues (*p* = 0.0005) (Figures 1(a), 1(b), 2(a), and 2(b)).

**Figure 3:**
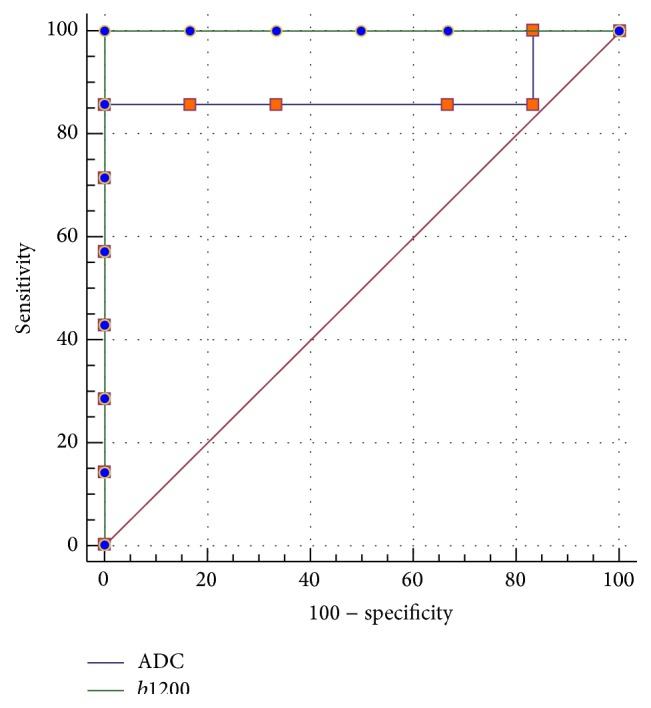
The ROC curve analysis of DW imaging for *b* value 1200 and ADC.

The ROC curve analysis proved very high sensitivity and specificity of DW methods: from 89% (sensitivity) and 85% (specificity) for ADC to 100% (sensitivity) and 100% (specificity) for *b* value 1200, respectively (Figure 3).

## 4. Discussion

Magnetic resonance imaging is regarded as an accurate technique for the detection and characterization of peritoneal spread of abdominal and pelvic malignancies. Advances in MRI technology, such as introduction of high-performance gradient systems, parallel imaging, or increased field homogeneity, resulted in improvement of quality of several MR techniques, including diffusion-weighted imaging. According to published reports this method could be also implemented for the detection of metastatic peritoneal disease in patients with malignant ovarian lesions [10].

At present, CT is the method of choice in preoperative evaluation of ovarian cancer and has been proved an accurate technique for predicting the results of cytoreduction in bulky disease [8]. In cases of “probably” early-stage ovarian cancer, limited only to ovarian mass, peritoneal implants are small and single, making the preoperative diagnosis challenging. CT is often not capable of reliably identifying small implants (with maximum diameter less than 5 mm) on the mesentery, bowel serosa, or peritoneum, especially in the absence of ascites [8, 11]. When diagnosed by MRI, these small lesions are usually better seen on DW images than on standard T1 and T2-weighted images [12].

Sala et al. in 2012 described the group of 22 ovarian cancer patients in which the men ADC for peritoneal metastases was lower than that of omental (*p* = 0.006) and ovarian mass (*p* = 0.015) [10].

DW imaging, especially when quantitative analysis is performed, has several limitations. The acquisition as well as analysis of DW images is not standardized. The choice of* b* values (number, range, and the first* b* value) and the method for ADC calculation (monoexponential versus biexponential model) have important implication for calculated ADC values of analyzed lesions. On the expense of prolonged imaging time, we implemented 8* b* values (0, 50, 100, 150, 200, 400, 800, and 1200 s/mm^2^) aiming to obtain more reliable ADC values than with the use of less* b* values. However, alternatively, we cannot exclude that monoexponential model applied in this study for ADC calculation could be less accurate than biexponential model [13].

One of the important limitations of this study is small number of patients. Therefore our results have to be regarded as preliminary in terms of sensitivity and specificity of DW imaging for the detection of small peritoneal implants and should be confirmed on larger group of patients.

## 5. Conclusions

The results of this preliminary study confirmed that implementation of DW imaging has potential for the detection of small peritoneal metastatic implants, which is very important especially in patients with potentially early malignant ovarian masses. We presume that implementation of DW imaging of the abdomen and pelvis may provide important, supplementary information regarding extension of neoplastic disease and influence patient's treatment.

## Figures and Tables

**Table 1:** Parameters of applied MR sequences.

Parameter	T2 TSE	T2 TSE Fat-Sat	DW EPI	T2 TIRM	3D T1 GRE
Repetition time (ms)	4250	2110	3800	6100	3,05
Echo time (ms)	117	123	73	39	1,13
Flip angle (deg.)	137	150	90	150	10
Turbo factor	51	51	—	9	—
EPI factor	—	—	96	—	—
iPAT factor	—	2	2	—	2
Plane	Axial	Axial	Axial	Axial	Axial
Number of signal averages	1	1	4	1	1
Field of view, FOV (mm)	360	360	360	360	360
Rectangular FOV (%)	75	100	75	75	75
Matrix	384 × 512	256 × 256	96 × 128	288 × 384	156 × 288
Slice thickness (mm)	5	5	6	5	3
Respiratory triggering	No	Yes	No	No	No
Breath-hold	No	Yes	No	No	No

**Table 2:** Locations of the extraovarian lesions.

Location	*N*—total 18
Hilus of the liver	2 (one false positive)
Cecum	2
Omental sac	1
Omentum	4
Douglas pouch	4
Diaphragm	2
Capsule of the liver	1
Transverse colon	1
Mesentery	1
